# Interpreting Anxiety Disorders From the Perspective of Interoceptive Computational Models

**DOI:** 10.1002/brb3.71019

**Published:** 2025-11-26

**Authors:** Zihan Lin, Shiqi Liao, Shasha Zhu, Yuqing Zhao, Wen‐Jing Yan, Ke Jiang, Kaiyu Qiu

**Affiliations:** ^1^ Psychological Counseling and Treatment Center The Second Hospital of Jinhua Jinhua China; ^2^ School of Mental Health Wenzhou Medical University Wenzhou China; ^3^ The Affiliated Kangning Hospital of Wenzhou Medical University Wenzhou China; ^4^ Key Laboratory of Alzheimer's Disease of Zhejiang Province, Institute of Aging Wenzhou Medical University Wenzhou China; ^5^ Applied Psychology Research Center, College of Psychiatry Wenzhou Medical University (Ningbo) Ningbo China; ^6^ School of Teacher Education Lishui University Lishui China; ^7^ School of Marxism Lishui University Lishui China

**Keywords:** anxiety disorders, computational models, interoception, predictive processing

## Abstract

**Introduction:**

Interoception—the nervous system's sensing, integration, and interpretation of internal bodily signals—facilitates the dynamic alignment of internal states with external environments. Predictive processing theory posits that this alignment arises from iterative comparisons between predicted and actual sensory inputs. Persistent mismatches in these computations may drive interoceptive dysfunction, a core mechanism implicated in anxiety disorders. Despite advances, a significant gap remains in linking computational models of interoceptive dysregulation to clinical interventions. This review synthesizes evidence to propose an interoceptive computational framework to bridge mechanistic insights with therapeutic innovation for anxiety.

**Methods:**

This review synthesizes evidence from computational psychiatry, neurophysiology, and clinical studies to model anxiety as a disorder of interoceptive prediction. We integrate predictive coding mechanisms underlying threat perception with the potential of experimental paradigms and bidirectional modulation strategies for intervention.

**Results:**

Anxiety pathophysiology is driven by hyperprecise threat priors and context rigidity, which amplify interoceptive prediction errors. These computational failures manifest as exaggerated defensive responses, cognitive biases, and maladaptive behaviors. Integrating computational modeling with targeted interventions, such as interoceptive exposure grounded in Bayesian belief updating, improves diagnostic precision and therapeutic outcomes.

**Conclusion:**

By bridging computational theories of interoceptive dysregulation with clinical practice, this framework advances a multidimensional approach to anxiety disorders. Future research should prioritize perturbed‐prior experiments and hybrid interventions to optimize personalized treatment. Such integration holds transformative potential for precision psychiatry, addressing both neural computations and embodied experiences in anxiety.

## Graphical abstract

Pathological anxiety stems from computational failures in interoception, where rigid threat beliefs amplify prediction errors. This review outlines how these mechanisms sustain maladaptive behaviors and cognitive biases. We synthesize a path forward by integrating computational models with targeted interventions to recalibrate this dysregulated process for improved clinical outcomes.

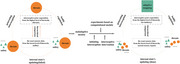

## 1 Introduction

Anxiety represents an adaptive response to environmental stressors (DeMartini et al. 2019, Kalin 2020). However, when excessive or prolonged, it can impair cognitive, social, and physiological functioning, potentially developing into a clinical anxiety disorder (DeMartini et al. 2019, Jalal et al. 2020). A primary diagnostic challenge lies in objectively distinguishing adaptive from pathological anxiety. Currently, this distinction relies heavily on clinician judgment (Crocq 2015). Despite the operational criteria for anxiety disorders provided by DSM‐5 and ICD‐11 (e.g., duration, severity, and functional impairment) (Penninx et al. 2021), they lack mechanistic biomarkers to validate these clinical thresholds. The development of such biomarkers is hampered by etiological complexity, clinical heterogeneity, high comorbidity rates, and poor specificity (Łoś and Waszkiewicz 2021). Furthermore, the frequent comorbidity of anxiety with other mental disorders (Kalin 2020, Bonaz et al. 2021, Szuhany and Simon 2022) adds another layer of diagnostic complexity, often complicating treatment planning (Penninx et al. 2021, Alonso et al. 2018, Brewer et al. 2021).

Anxiety is considered a kind of interoceptive disorder (Abend 2023). This theoretical shift offers a promising path forward. Specifically, quantifying interoceptive abilities could inform the development of more precise, biologically grounded diagnostic criteria (Brewer et al. 2021). Moreover, interventions aimed at improving interoceptive accuracy may present novel mechanisms for treatment (Sugawara et al. 2020, Quadt et al. 2021).

This review first synthesizes the definitions of interoception, its physiological foundations, and its computational modeling hypotheses. Building upon previous studies, we leverage interoceptive computational models to: (1) explain interoceptive dysfunction mechanisms, (2) decode plausible anxiety disorder pathogenesis through interoceptive signatures, and (3) propose testable hypotheses for future research and suggest translational pathways.

## 2 The Definition of Interoception and its Relationship With Physical and Psychological Health

The precise conceptualization of interoception remains controversial, particularly regarding the scope of the “internal states” it encompasses (Toussaint et al. 2024). Historically, research focused narrowly on visceral sensations. However, contemporary definitions have expanded to include a broader range of signals, such as those from affective touch (e.g., hugs, kisses) (Khalsa and Lapidus 2016, Chen et al. 2021, Yang and Zhu 2022).

From a neuroscientific perspective, interoceptive signals are conveyed via the spinal and trigeminal dorsal horn or cranial nerves (e.g., the vagus) to the nucleus of the solitary tract, ultimately projecting to the anterior insula and anterior cingulate cortex (Cameron 2001, Craig 2002, Olausson et al. 2002, Critchley and Harrison 2013). These signals originate from diverse tissues, including skin, bones, and smooth muscles. Behaviorally, interoception is not a unitary process but a foundational component of autonomic reflexes, homeostatic drives, affective experiences, and cognitive representations of the bodily self (Khalsa et al. 2018). Bonaz et al. further postulate that the continuity of interoceptive processing is fundamental to the neurobiological structure of self‐awareness (2021). Thus, interoception can be understood as a fundamental mind‐body integration process that is crucial for maintaining homeostasis, regulating emotional balance, and ensuring overall organismal adaptability (Khalsa et al. 2018).

Traditional perspectives posit that interoception follows a unidirectional pathway (Berntson and Khalsa 2021). Enhancing interoceptive skills is often viewed as improving the ability to recognize and interpret bodily states, a process crucial for maintaining homeostasis (Fotopoulou and Tsakiris 2017). When homeostasis is disrupted, bodily signals are sent to the brain, triggering internal sensations that prompt regulatory responses (Paulus et al. 2019). According to this unidirectional model, interoception is thus characterized as a passive, adaptive response to external stimuli. To survive, an organism must balance environmental adaptation with internal stability (Petzschner et al. 2021). However, homeostatic reflex mechanisms face inherent constraints: in non‐stationary environments, rigid reflex arcs may prove maladaptive. Moreover, afferent signaling is often contaminated by “noise,” impairing signal discrimination (Kavcıoğlu et al. 2021). Consequently, the unidirectional model appears insufficient to explain interoceptive complexity, necessitating more robust adaptive frameworks.

Therefore, a growing number of researchers have proposed various computational models to explain interoceptive adaptability. Notable examples include the allostatic self‐efficacy model within a Bayesian hierarchical framework (Stephan et al. 2016), computational models formed on active inference (Paulus et al. 2019), and the sensory‐control loop model (Bonaz et al. 2021, Brewer et al. 2021, Petzschner et al. 2021), all of which are discussed in detail in Section 3.2 (Table 1).  These frameworks conceptualize interoception as the process in which the nervous system integrates and interprets internal signals to construct a dynamic representation of the body's internal state, as well as the “mapping” between the body and the external environment (Paulus et al. 2019). Rooted in predictive processing, these models suggest that organisms incessantly update predictions by matching them iteratively with actual inputs, refining the internal state representation. (Paulus et al. 2019, Stephan et al. 2016, Barrett and Simmons 2015, Paulus and Stein 2010, Aupperle et al. 2014).

**TABLE 1:** Comparison of interoceptive computational models.

Aspects		Models		
		Allostatic Self‐efficacy (Stephan et al. 2016)	Active inference (Paulus et al. 2019)	Sensory‐control loop (Petzschner et al. 2021)
Differences	Core thesis	The thesis posits that metacognitive failures ininteroceptive prediction are the root cause of fatigue and depression.	Models how dysregulated prediction‐error signaling sustains anxiety and depression.	Provides a unifying framework for interoception, body regulation, and forecasting.
	Model specificity	High‐level: Focuses on a metacognitive layer evaluating the brain's confidence in its allostatic self‐efficacy.	Mid‐level: Details the mechanisms by which faulty precision‐weighting of errors leads to chronic interoceptive mismatch.	System‐level: Describes a functional loop (sense–regulate–forecast) linking bodily perception with adaptive action.
Similarities		Core principle: All models conceptualize interoception as a hierarchical Bayesian process of prediction‐error minimization. Mathematical basis: Each is grounded in predictive coding and Bayes’ theorem, describing how priors and sensory evidence are integrated. Neural substrate: All converge on a cortico‐limbic hierarchy (insula, ACC, PFC) for processing ascending interoceptive signals and descending predictions.		

Dysregulation in interoceptive signal integration can disrupt homeostasis and is strongly associated with negative emotional responses and psychosomatic disorders (Bonaz et al. 2021, Brewer et al. 2021, Khalsa and Lapidus 2016, Khalsa et al. 2018, Barrett and Simmons 2015, Murphy et al. 2017). Specifically, dysfunctional interoception has been implicated in neurological disorders (both structural and functional) (Ricciardi et al. 2016, Koreki et al. 2020), psychosomatic diseases (Icenhour et al. 2017, Mayer et al. 2019, Fournier et al. 2020), and psychiatric disorders (including comorbidities) (Bonaz et al. 2021, Quadt et al. 2021, Stephan et al. 2016, Garfinkel et al. 2016, Palser et al. 2018, Kutscheidt et al. 2019, Noda et al. 2022, Adams et al. 2022, Xiang et al. 2022). These conditions may stem from persistent deviations in real‐time interoceptive representation, reflecting impaired updating of perceptual mappings (Paulus et al. 2019).

## 3 The Interoceptive Computational Models

Computation theory aims to construct mathematically defined models that precisely explain adaptive response mechanisms under varying external stimuli (Khalsa et al. 2018). Interoceptive computational models are particularly suited to elucidate organismal adaptation for the following reasons:

For survival, organisms must adapt to ever‐changing environments. The body mediates this environmental information exchange (Su and Ye 2021), requiring receptors to flexibly align internal and external signals while distinguishing past from present states to guide adaptive responses (Møbjerg and Neves 2021, Tsukahara et al. 2021). As Kleckner et al. emphasize, interoception is a temporally extended, survival‐oriented process linking bodily states to past memories, current contexts, and future needs (2017). Interoceptively guided actions maintain organism–environment homeostasis by dynamically renewing equilibrium and shaping future sensory inputs, forming a self‐sustaining sensory–control loop. This loop is formally conceptualized within a hierarchical Bayesian interoceptive computational framework, where actions are guided by probabilistic inferences aimed at minimizing prediction errors and maintaining homeostatic equilibrium. (Petzschner et al. 2021). Such interactions are inherently proactive and flexible.

Crucially, interoceptive computational theory underscores the significant role of the body in cognitive processes, advancing the view that meaning is generated by representational action (Su and Ye 2021, Ye and Yang 2018) and brain‐body‐environment coupling (Su and Ye 2021, Ye et al. 2021, Ye 2022). Consequently, interoceptive computational models become the prevailing framework for analyzing adaptive processes.

### 3.1 The Neural Basis of the Interoceptive Computational Models

From a neurophysiological perspective, an organism's interoception reflects the dynamic integration of physiological signals (e.g., body temperature, blood pressure, bladder pressure, heart rate, osmotic balance) (Craig 2002, Velten et al. 2018). Specialized receptors (chemo‐, mechano‐, and osmoreceptors) detect these signals and transmit them via bottom‐up pathways (Berntson and Khalsa 2021). Peripheral inputs are initially relayed to the nucleus of the solitary tract (Holt et al. 2019), followed by top‐down modulation of visceral states. These descending signals engage autonomic, endocrine, and immune pathways to regulate hormone release, autonomic activity, and homeostatic reflexes (Berntson and Khalsa 2021, Petzschner et al. 2021). Mouse studies demonstrate that insular cortex regions encoding hunger/thirst dynamically update satiety predictions based on environmental cues (e.g., food/water availability), illustrating context‐dependent top‐down regulation of interoceptive coding (Livneh et al. 2020). Similarly, the hypothalamus integrates reactive and predictive control within the endocrine system (Burdakov 2019). Tan et al. identified the dorsal anterior insula as a convergent hub for interoception, decision‐making, and emotion regulation (2022). This region co‐activated with other salience network nodes (e.g., anterior cingulate cortex, thalamus), supporting a hierarchical model in which the insula integrates ascending bodily signals for higher‐order cognitive processes to minimize prediction error.

Although the bottom‐up and top‐down pathways serve distinct functions, their complex interactions complicate the mapping of specific afferent‐efferent circuits in interoception (Jung Hoon et al. 2020). According to Berntson and Khalsa, bidirectional signaling is central to adaptive interoceptive processing, with pathway integration being key to understanding organismal adaptation. This duality—where interoception both generates predictions and responds to prediction errors—aligns with active inference principles. Specifically, organisms minimize prediction errors through either perceptual updating (revising predictions) or active inference (altering sensory inputs via action) (2021). How this dual process works from the perspective of computational models is to be discussed in detail below.

### 3.2 Interoception Within the Sensory‐Control Loop Model

**FIGURE 1:**
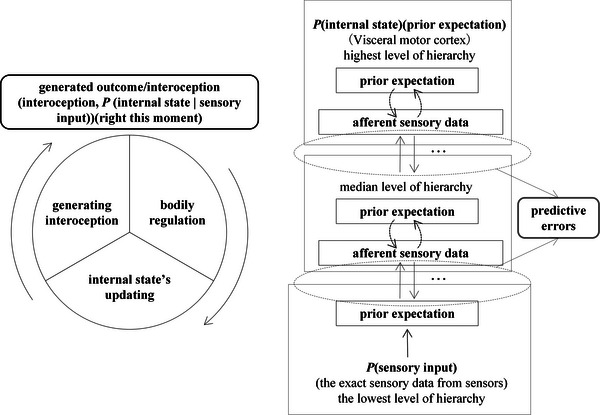
The sensory‐control loop and the generating of interoception (created on the basis of (Petzschner et al. 2021). This Bayesian hierarchical process—from generating interoceptive signals to executing adaptive responses and updating internal models—forms a comprehensive, real‐time representation of the body's physiological state. This closed loop enables precise alignment with the external environment, facilitating optimal organismal adaptation.

Stephan et al. proposed the allostatic self‐efficacy model within a Bayesian framework, theorizing fatigue and depression as emergent metacognitive disturbances from interoceptive prediction failure (2016). This metacognitive theory of illness emphasizes that higher‐order beliefs about the brain's regulatory capacity (allostatic self‐efficacy) mediate the fatigue‐to‐depression transition. Complementing this, Paulus et al. identified two mechanisms in interoceptive dysfunction through an active inference model: hyperprecise priors (overestimated certain expectations) and context rigidity (impaired adjustment to environmental changes) (2019). Expanding the theoretical scope, Petzschner et al.’s sensory‐control loop model unified interoceptive computation, body regulation, and predictive forecasting across brain‐body disorders (2021), which comprises three components: (1) a generative model constructing interoceptive representations, (2) a regulatory model guiding adaptive responses, and (3) a predictive model updating internal states (Figure 1).

The generative model, as the loop's first stage, infers bodily states from sensory signals by integrating implicit and explicit knowledge. Under Bayesian principles, the brain weights noisy sensory inputs against prior expectations to generate optimal inferences. Mechanistically, higher cortical regions send predictions to lower‐level areas, which compare them with ascending bodily signals. When predictions match sensory inputs (minimal prediction error), receptor inputs are validated as accurate. Discrepancies trigger dual adjustments: predictions are revised, and peripheral signals are modulated (e.g., via autonomic pathways) to restore homeostasis. Uncorrected errors may propagate maladaptive interoception (Petzschner et al. 2021). Thus, interoception is a continuous process whereby predictions are aligned with sensory evidence to minimize error. This anticipatory inference explains why perceived bodily states (e.g., blood pressure) often deviate from physiological measurements—prior expectations dominate when sensory precision is low. For instance, hypertensive patients rarely subjectively estimate extreme pressures (e.g., > 180/110 mmHg), demonstrating top‐down modulation by pre‐existing knowledge (Petzschner et al. 2021, Hulme et al. 2019).

After minimizing errors between predictive signals and sensory inputs (prediction errors), organisms must enact adaptive behaviors to maintain homeostasis. For instance, they may add clothing in response to cold or cease eating upon satiety. Paulus et al. frame this regulatory phase as active inference—a process central to interoceptive generation. Building on this, Petzschner et al. and Tschantz et al. (2021, 2022) formalize how predictive encoding steers action selection toward homeostatically optimal states. Through such actions, individuals refine predictions, further reducing errors and aligning actual bodily states with expected conditions (Paulus et al. 2019, Aupperle et al. 2014, Seth and Friston 2016, Allen et al. 2023).

The predictive updating phase completes the loop, where the organism revises its internal state expectations based on environmental inputs and enacted behaviors. This mechanism is exemplified by subfornical organ (SFO) thirst neurons, which integrate orosensory cues during consumption with real‐time blood metrics to forecast fluid balance changes, thereby guiding preemptive adjustments (Zimmerman et al. 2016). For instance, during alcohol intake, interoceptive prediction errors may trigger drinking cessation when blood osmolarity disruption is anticipated—an adaptive response preventing physiological instability (e.g., dizziness). Subsequent alcohol concentration stabilization updates the interoceptive prior, while continued drinking would generate alternative predictive states, each driving further updates.

Within this framework, interoception serves dual computational roles: a continuous inference process and a perceptual outcome. The iterative predict‐update‐act triad sustains core physiological functions, with timely interoceptive updates being critical for environmental adaptation.

### 3.3 To Interpret Psychiatric Disorders From Interoceptive Computational Models

As noted above, when the interoception loop functions optimally, organisms accurately integrate and interpret bodily signals—a process termed interoceptive inference that continuously updates self‐representation (Khalsa et al. 2018, Paulus et al. 2019). Disruptions distort this process, yielding chronic biases that impair adaptability.

Within the active inference framework (Paulus et al. 2019), interoceptive percepts reflect a Bayesian “best estimate” derived from prior expectations and sensory evidence (Stephan et al. 2016, Barrett and Simmons 2015, Paulus and Stein 2010, Aupperle et al. 2014, Friston et al. 2009). This “best estimate” is shaped by both genetic predispositions and experiential learning (Petzschner et al. 2021), representing an evolutionary strategy for survival (Tschantz et al. 2022). The generative models established during the initial phase of the sensory‐control loop, alongside subsequent actions, support this framework (Morville et al. 2018). However, the “best estimate” becomes maladaptive when dysfunctional interoception perpetuates erroneous feedback loops.

## 4 Interoceptive Dysfunction and Anxiety Disorders

### 4.1 Interpreting Anxiety Disorders Within the Sensory‐Control Loop Model

Research suggests that disruptions in interoceptive processing may foster a cognitive bias toward threat perception, impair the accurate sensing of bodily states, elevate anxiety, increase allostatic load, and contribute to mental health disorders (Paulus and Stein 2006, Hakamata et al. 2010, Sterling 2014, Peters et al. 2017, Van den Bergh et al. 2017). Within dynamic environments, individuals with persistent anxiety are particularly susceptible to the dominance of prior expectations over actual interoceptive signals (Paulus et al. 2019). Furthermore, studies have found that impaired interoceptive abilities are connected to the development of cognitive bias (Smith et al. 2020, Büttiker et al. 2021, Verdonk et al. 2024). Such cognitive bias is strongly correlated with persistent anxiety symptoms (Lemente et al. 2024). Critically, anxiety is not a static condition but a dynamic state where symptoms fluctuate over time, making their specific manifestation and timing difficult to predict (Abend 2023, Lapate and Heller 2020). Therefore, characterizing anxiety necessitates a dynamic framework, which the aforementioned computational models provide by explaining how dysfunctional Bayesian updating perpetuates symptom variability.

**FIGURE 2:**
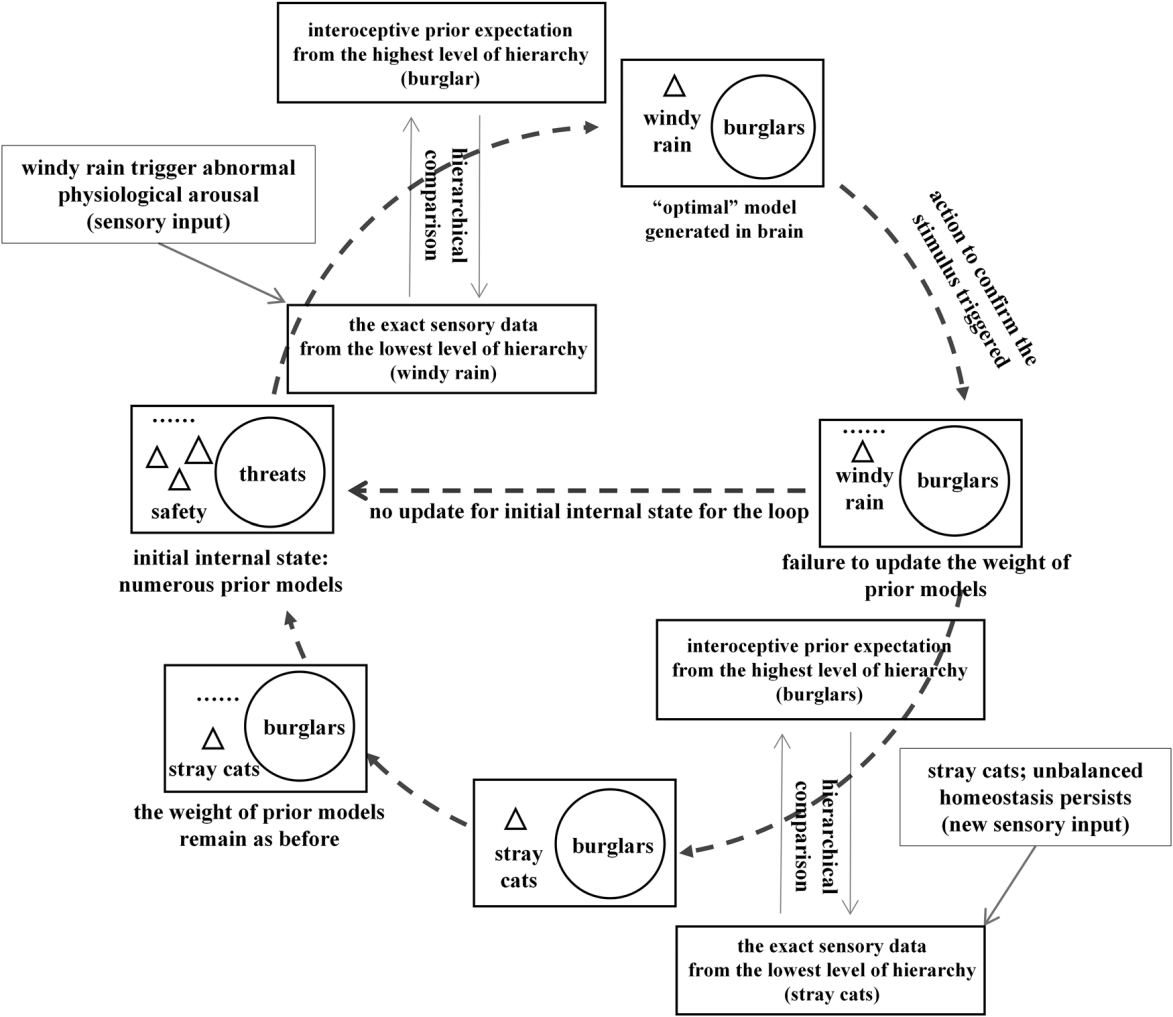
How active inference in the sensory‐control loop interprets the mechanism of interoceptive dysfunction for individuals with anxiety—hyperprecise threat priors and context rigidity (created on the basis of (Paulus et al. 2019) and (Petzschner et al. 2021). In individuals with maladaptive anxiety, initial perception is biased toward threat‐predictive models, a tendency reinforced by higher‐order brain functions. Through hierarchical processing, even benign stimuli (e.g., the sound of wind and rain) are interpreted as potential threats. Due to impaired weighting of prior predictions, internal models fail to update appropriately, propagating errors into subsequent perceptual cycles. For example, when the weather clears and a new stimulus appears—such as a stray cat moving quickly through bushes—these individuals persist in applying threat‐based models. This results in a persistently biased “best fit” perception throughout the hierarchy, sustaining anxiety and perpetuating faulty threat detection.

Interoceptive dysfunction primarily manifests through two computational anomalies: hyperprecise priors and context rigidity (Paulus et al. 2019). Hyperprecise priors reflect an overestimation of a certain model during interoceptive inference, leading to exaggerated belief states. Context rigidity describes the failure to flexibly update these priors in response to changing internal or external cues (Figure 2). Consequently, prediction errors persist throughout the sensory‐control loop, disrupting homeostasis and perpetuating dysfunction (Paulus et al. 2019, Tschantz et al. 2022, Smith et al. 2020). This framework aligns with clinical observations of anxiety as a continuum of aberrant defensive responses. Abend conceptualizes anxiety as characterized by heightened cognitive vigilance and physiological arousal—symptoms indicative of a malfunctioning defense system (2023). Similarly, Richter et al. note that panic disorder and agoraphobia represent an escalation of defensive responses to internal threat cues, with patients exhibiting heightened interoceptive sensitivity (2021). These perceived threats often originate from misrepresented bodily signals, such as arrhythmias or hypoxia (Khalsa et al. 2018, Benke et al. 2021). Therefore, in the context of anxiety, this mechanism of “hyperprecise priors” appears to specifically manifest as “hyperprecise threat priors,” whereby individuals disproportionately weigh expectations of danger or harm.

**FIGURE 3:**
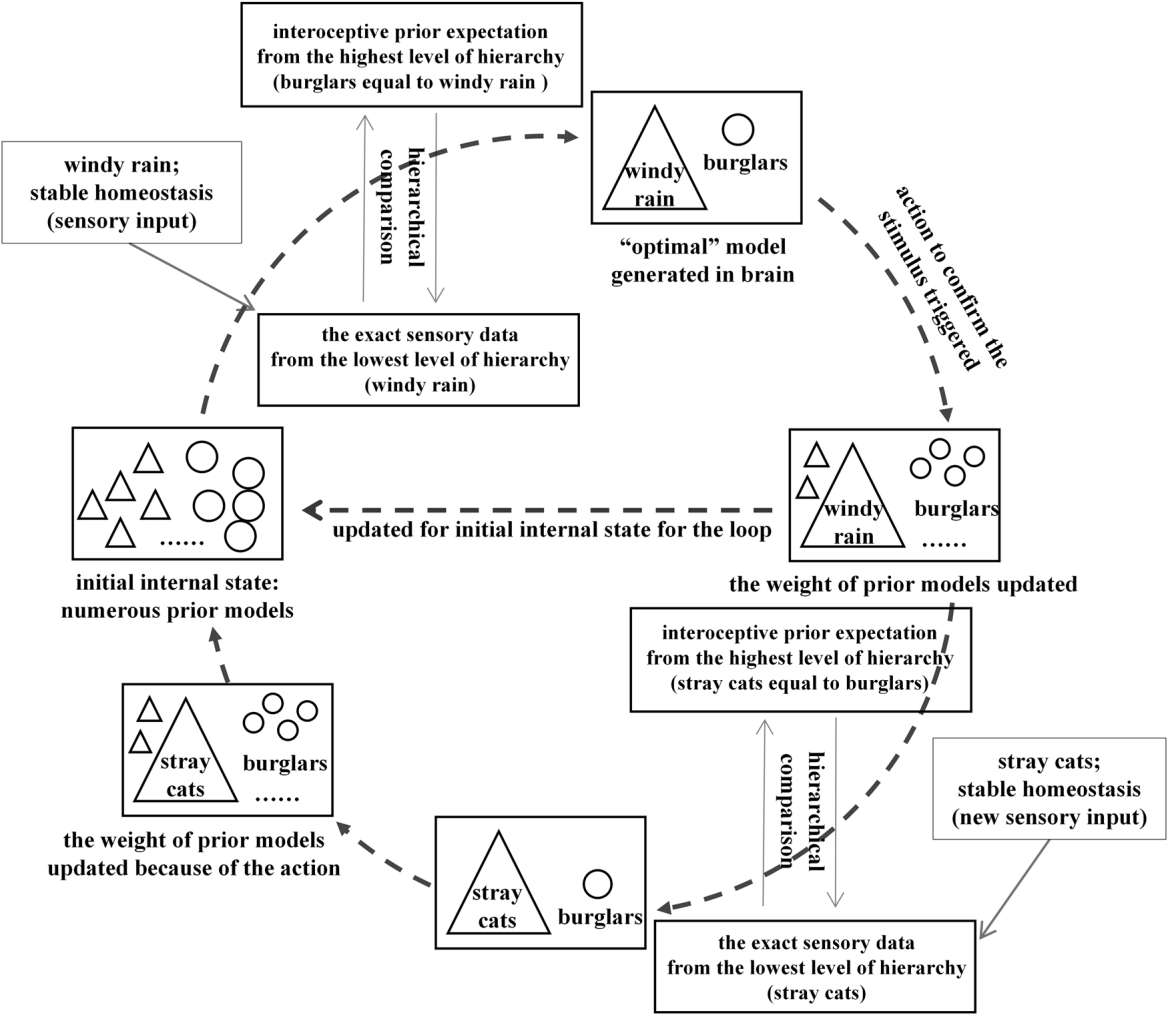
How active inference in the sensory‐control loop interprets the mechanism of well‐functioned interoception (created on the basis of (Paulus et al. 2019) and (Petzschner et al. 2021). In the initial phase of perceptual inference, individuals balance predictive models of threat and safety. Upon hearing the sound of wind and rain, top‐down expectations from higher cortical areas engage in hierarchical comparison with bottom‐up sensory signals. A best‐fitting model—in this case, “wind and rain”—is selected to minimize prediction error, leading to an action that further confirms the stimulus and updates the prior weighting of the predictive model. This revised internal state then informs the next cycle of perception. When the environment changes—for example, when a stray cat moves quickly through nearby bushes—new sensory input prompts iterative matching against internal predictions. The perceptual system converges on the “stray cat” as the most likely cause, and the internal model is updated accordingly. Throughout this process, individuals do not over‐prioritize threat‐related expectations despite sensory uncertainty; instead, they adaptively adjust their internal states.

Consider a stormy night as an example. An individual with maladaptive anxiety may disproportionately weight threat‐related priors upon hearing wind and rain against a window, triggering a heightened defensive physiological response (Paulus et al. 2019). They might misinterpret this sound as an intruder. Crucially, even after receiving corrective sensory evidence (e.g., visually confirming it is only the wind), context rigidity prevents prior belief updating. A similar stimulus will likely reinstate the same threat response (e.g., “Perhaps it is an intruder this time”). This failure to update beliefs generates persistent prediction errors, sustaining vigilance and symptoms like tachycardia, tachypnea, and motor agitation. Later, a new stimulus (e.g., a noise from bushes after the storm) should prompt a new assessment. However, hyperprecise threat priors continue to dominate perception, overriding sensory evidence and preventing adaptive responses (Tschantz et al. 2022). This inflexible adherence to maladaptive models amplifies anxiety and impedes behavioral adaptability. In contrast, neurotypical individuals can flexibly evaluate and weigh competing expectations (e.g., “It's probably just a cat”), thereby minimizing prediction error and stabilizing their physiological state (Paulus et al. 2019) (see Figure 3).

When interoceptive functioning is optimal, an organism effectively manages physiological deviations from a homeostatic set point or adaptively redefines this set point allostatically to enhance survival (McEwen 2017). In anxiety disorders, this flexibility is lost due to hyperprecise threat priors (rigid, threat‐biased expectations) and context rigidity (an inability to update these expectations). These misrepresentations are processed through dysregulated autonomic pathways—engaging the thalamus, limbic system, locus coeruleus, and hippocampus—which triggers neurochemical responses such as increased norepinephrine release. Upon reaching the cerebral cortex, these signals, filtered through maladaptive priors, distort metacognition, culminating in panic‐driven emotional and physiological symptoms (Nayok et al. 2023). Critically, the core mechanism sustaining this cycle is the failure to reduce prediction errors through sensory evidence. The brain's inability to desensitize its hyper‐precise threat models via real‐time alignment with actual bodily inputs (Paulus and Stein 2006, Mathys 2011) results in a state of chronic anxiety. Minor physiological fluctuations are catastrophically misinterpreted, maintaining heightened vigilance and perpetuating symptoms (Szuhany and Simon 2022).

Thus, interoceptive impairment diminishes the ability to accurately perceive bodily states, creating a persistent cognitive bias within the sensory‐control loop. This bias primes individuals with anxiety to anticipate threat in neutral stimuli, triggering disproportionate responses that maintain the disorder.

### 4.2 Interoceptive Dysfunction of Anxiety Disorders

The interoceptive sensory‐control loop model provides a framework for understanding dysfunction in anxiety disorders, which is supported by empirical evidence of aberrant threat reactivity. For instance, research demonstrates that individuals with Generalized Anxiety Disorder (GAD) exhibit hypersensitive responses to interoceptive challenges.

Teed et al. found that women with GAD, compared to healthy controls, showed heightened autonomic reactivity (e.g., increased heart rate) and subjective anxiety to low‐dose adrenergic stimuli, coupled with delayed neural activation in the ventromedial prefrontal cortex (vmPFC) (2022). Complementing this, Verdonk et al. reported that GAD patients had significantly higher heart‐evoked potential (HEP) amplitudes—a neural correlate of interoceptive prediction error (the discrepancy between predicted and actual cardiac signals)—even in response to neutral saline injections (2024). This consistent amplification of prediction errors suggests an underlying computational failure. Also, Harrison et al. found that anxiety is less tied to low‐level perceptual thresholds than to higher‐order beliefs about bodily states—especially catastrophizing interpretations of interoceptive signals. Individuals with moderate anxiety show heightened anterior insula reactivity to threat‐related respiratory predictions, consistent with distorted precision weighting of interoceptive information (2021). This may reflect overweighting of prediction errors for threatening sensations, perpetuating maladaptive body‐brain communication. It seems that GAD could be characterized by hyperprecise threat priors—overly rigid and inflated expectations of cardiac threat. This may manifest as a compensatory hyper‐vigilance (a hyper‐defensive response (Richter et al. 2021)) that paradoxically increases prediction errors, as the brain generates exaggerated top‐down predictions that are continually disconfirmed by bottom‐up reality (Paulus et al. 2019, Petzschner et al. 2021, Verdonk et al. 2024, Petzschner et al. 2019). This malfunction perpetuates a cycle of anxiety and aberrant physiological arousal.

The diagnostic criteria for anxiety disorders encompass both physiological symptoms (e.g., tachycardia, tachypnea, sweating, muscle tension) (Szuhany and Simon 2022) and psychological ones (e.g., unease, irritability, difficulty concentrating, depersonalization) (Jalal et al. 2020, World Health Organization (WHO) 2019), many of which are linked to interoceptive processing (Khalsa et al. 2018). Individuals with anxiety often report severely heightened physiological arousal (Verdonk et al. 2024, Teed et al. 2022, Kircanski et al. 2018, LeDoux and Daw 2018, Joyal et al. 2019), describing symptoms indicative of sympathetic hyperactivity. However, objective physiological assessments frequently reveal a discrepancy between this subjective distress and measurable bodily states (Adams et al. 2022, Verdonk et al. 2024, Teed et al. 2022, Makovac et al. 2019). This mismatch between perceived and actual physiology highlights a core interoceptive dysfunction: a failure to accurately interpret and integrate bodily signals into subjective experience (Connell et al. 2018, Zhang and Chen 2021). Consequently, common anxiety symptoms may originate from maladaptive interoceptive inference, where faulty prior beliefs dominate sensory evidence (Nayok et al. 2023), making interoceptive disruption a potential hallmark of these disorders (Khalsa and Lapidus 2016, Adams et al. 2022).

A developmental pathway to anxiety may originate in early temperament. Allen et al. suggest that behaviorally inhibited infants exhibit heightened physiological reactivity to novelty, a trait that persists and escalates into attentional and interoceptive biases toward perceived threat (2023, Degnan and Fox 2007). This is reinforced over time as negative perceptions of arousal amplify threat responses and anxiety risk (Allen et al. 2023). This foundational dysfunction manifests in adulthood as a core inability to accurately process bodily signals. Stein's concept of anxiety “false alarms” (2006), captures this well: benign internal sensations are mistakenly interpreted as threatening stimuli requiring avoidance (Paulus and Stein 2010, Domschke et al. 2010). This imprecision generates profound intolerance of uncertainty—a hallmark of pathological anxiety where individuals hold negative beliefs about ambiguous threats (MacLeod et al. 2019, Yamamori and Robinson 2023). These behavioral phenomena are also supported by neural evidence. Li et al. demonstrated that patients with GAD exhibit aberrant brain network responses even under neutral conditions, reflecting a failure to distinguish safety from threat and to manage uncertainty (2020). Given the studies above, we propose that the heightened physiological response in individuals with pathological anxiety may be attributed to biased certainty of perceived threats, which triggers intensified defensive responses. Additionally, this heightened sensitivity may originate from experiences during one's infancy.

### 4.3 Interim Synthesis and Hypotheses

Synthesizing the extant literature, we posit that anxiety disorders are fundamentally disorders of interoceptive inference, characterized by a failure to accurately integrate bodily signals with environmental context. Based on this synthesis, we propose the following hypotheses:

1. **The Precision Hypothesis**: Pathological anxiety arises from a pathological increase in the certainty assigned to threat‐related prior beliefs. This leads to a cognitive bias wherein ambiguous interoceptive and exteroceptive stimuli are catastrophically misinterpreted, triggering disproportionate defensive responses (e.g., heightened physiological arousal, catastrophic cognition).
2. **The Developmental Trajectory Hypothesis**: The establishment of these hyperprecise threat priors is facilitated by early‐life factors (e.g., behavioral inhibition temperament), where heritable predispositions and learned experiences reinforce negative associations with internal bodily sensations and environmental uncertainty.
3. **The Cycle of Dysregulation Hypothesis**: This initial deficit initiates a vicious cycle: biased priors generate persistent prediction errors, which the dysfunctional system fails to resolve through adaptive belief updating or action (context rigidity). This chronic mismatch further entrenches the faulty priors, worsening interoceptive impairment and solidifying the pathological anxiety state.

In essence, we hypothesize that while adaptive anxiety is a protective mechanism driven by efficient interoceptive monitoring, its pathological form emerges when the brain's predictive machinery becomes locked in a self‐perpetuating loop of false alarms and failed error correction to threats.

## 5 Future Perspectives on Integrated Application of the Interoceptive Computational Models to Anxiety Disorders

### 5.1 The Significance of Interoceptive Computational Models in Identifying Cognitive Biases in Anxiety Disorders

Wölk et al. used heartbeat counting tasks and the Iowa Gambling Task, reporting a tendency for patients with panic disorder to interpret interoceptive cues as threatening (2014). However, the interpretation of such findings is complicated by methodological debates. A primary critique of heartbeat perception tasks is that they may confound true interoceptive acuity with an individual's prior beliefs about their heart rate, rather than measuring a pure physiological ability (Paulus et al. 2019, Adams et al. 2022, Desmedt et al. 2018, Murphy et al. 2018, Zamariola et al. 2018). Consequently, studies investigating the anxiety‐interoception link with these tools have produced inconsistent results (Garfinkel et al. 2016, Ewing et al. 2017, Desmedt et al. 2020, Hickman et al. 2020), likely due to heterogeneous clinical samples and divergent anxiety measures (Adams et al. 2022). Furthermore, these tasks are not process‐pure; they engage multiple physiological and cognitive systems simultaneously, capturing only a fraction of the broader interoceptive spectrum (Baranauskas et al. 2017). Crucially, the overwhelming focus on cardiac perception has neglected other interoceptive domains, limiting the generalizability of existing findings on anxiety.

Despite methodological debates surrounding heartbeat perception tasks, evidence confirming that anxious individuals interpret interoceptive signals as threatening underscores a core cognitive bias. To move beyond static measures and capture this bias in dynamic contexts—while formally accounting for the role of prior expectations—computational modeling is essential. These models allow researchers to experimentally manipulate predictable versus uncertain sensory information to test how prior beliefs shape interoceptive inference (Khalsa et al. 2018, Paulus et al. 2019). Research found that individuals with pathological anxiety exhibit maladaptive learning patterns characterized by elevated learning rates for punishment or threat (Homan et al. 2019, Aylward et al. 2019, Pike and Robinson 2022). This dysfunction may manifest as hyperprecise threat priors by overweighting aversive cues or contextual rigidity—impaired adjustment of beliefs to sensory uncertainty, as demonstrated transdiagnostically by Smith et al. using the heartbeat tapping task (2020). These computational distinctions help dissect specific pathways to interoceptive dysfunction in anxiety.

Moreover, the comorbidity of anxiety and depression is highly prevalent in clinical practice (Bonaz et al. 2021), which complicates differential diagnosis. While both disorders involve impaired interoception (Brewer et al. 2021, Paulus et al. 2019) and a cognitive bias toward negative information (Noworyta et al. 2021), their underlying mechanisms may differ. Depression is frequently associated with diminished physiological and subjective responses to positive stimuli (e.g., anhedonia) (Bylsma et al. 2008, Sloan and Sandt 2010). Brewer et al. suggest this may reflect an active suppression of internal emotional signals, leading to emotional blunting (2021). This blunted affective reactivity in depression stands in direct contrast to the heightened sensitivity and exaggerated reactivity to threat and negative stimuli characteristic of anxiety disorders. Given its central role in emotional processing, interoceptive profiling shows promise as an objective biomarker for distinguishing these comorbid conditions.

### 5.2 Integrating the Interoceptive Computational Model With Clinical Work on Anxiety Disorders

Given that anxiety disorders are marked by significant disruptions in interoceptive processing, targeting this dysfunction represents a promising therapeutic avenue. This approach is grounded in the computational principle that the brain infers emotional states from bodily signals. As Paulus et al. argue, interventions can leverage bottom‐up pathways by directly manipulating interoceptive input (e.g., through respiration or thermoregulation). This helps recalibrate the brain's maladaptive body‐state representations, thereby reducing persistent prediction errors and promoting adaptive behavioral responses (2019).

Computational models are critical for elucidating the mechanisms of anxiety interventions, paving the way for precise treatments. For instance, modeling a drug's effect on approach‐avoidance behavior can dissect whether it works by enhancing reward sensitivity, diminishing punishment sensitivity, or both (Yamamori and Robinson 2023). This computational dissection is essential because anxiety‐related learning deficits are nuanced. Pike and Robinson's finding—that individuals with pathological anxiety show accelerated learning from punishment without heightened baseline punishment sensitivity (2022)—exemplifies this complexity. It suggests a dysfunction in belief updating rather than mere perceptual bias. Therefore, computational models can guide the development of interoceptive interventions that specifically target these maladaptive learning patterns. For example, a therapy could be designed to recalibrate the overweighting of punitive outcomes, thereby improving an individual's ability to navigate negative situations and enhancing overall treatment efficacy.

This computational approach directly informs the design of interoceptive interventions. The goal is to provide patients with volitional control over their maladaptive inference processes, primarily by recalibrating the precision weighting of interoceptive signals. For example, individuals can be trained to better recognize and accurately interpret bodily signals (e.g., distinguishing arousal from excitement rather than threat) (Duquette 2020). Alternatively, techniques can leverage innate physiological mechanisms to attenuate sensory evidence precision, such as using cutaneous stimulation (e.g., rubbing one's stomach) to dampen the perceived intensity of a stomachache (Smith et al. 2020, Kiverstein et al. 2019). This mechanistic focus on precision regulation underpins many body‐based therapies.

Recognized as an interoceptive intervention (Khalsa et al. 2018, Benke et al. 2021), Cognitive Behavioral Therapy (CBT), a gold‐standard treatment for GAD, effectively operates by facilitating this recalibration. (DeMartini et al. 2019). Through exposure and response prevention, CBT helps patients gradually update their overly precise threat priors by providing disconfirming sensory evidence, thereby establishing new, adaptive associations (Yamamori and Robinson 2023). Beyond CBT, a range of interventions target interoceptive inference through similar mechanisms: carbon dioxide‐assisted breathing training (Meuret et al. 2008, Meuret et al. 2018), mindfulness‐based stress reduction (MBSR), yoga, meditation, exercise‐based interventions (Farb et al. 2015, Wielgosz et al. 2019), and floatation‐REST therapy (Feinstein et al. 2018a, Feinstein et al. 2018b). These approaches integrate the body's internal and external processes, highlighting their beneficial effects on reducing pathological anxiety (Nayok et al. 2023).

Therefore, we propose: future research should leverage computational models to deconstruct these interventions within the sensory‐control loop framework. Specifically, studies must identify whether therapeutic efficacy stems from correcting misinterpretations at specific inference stages, improving belief updating flexibility, or enhancing the ability to act on new interoceptive insights. This precise mechanistic understanding is the key to developing targeted, effective, and personalized psychiatric treatments.

### 5.3 Prospects for Empirical Research on Interoception Based on the Computational Models Toward Anxiety Disorders

Given the challenges in directly quantifying interoception, a promising alternative is to probe its functional properties indirectly by examining how it is modulated by exteroceptive stimuli. This approach, grounded in computational models, involves perturbing the interoceptive system with controlled external tasks and measuring the resulting inference processes. Paulus et al. suggest that such paradigms can reveal fundamental differences in perceptual inference across clinical populations. For instance, by altering the association between an external cue and a subsequent bodily sensation, researchers can assess not only the accuracy of interoceptive perception but also the metacognitive confidence individuals have in their interoceptive judgments. This provides a window into higher‐order cognitive biases (Paulus et al. 2019). Experimental paradigms based on this principle are already being used to explore learning and decision‐making biases in anxiety (Yamamori and Robinson 2023). The ultimate goal of this research is to transition from mere assessment to targeted modulation—developing interventions that can systematically recalibrate maladaptive interoceptive inference to harness its therapeutic regulatory potential.

Neuroimaging research by Wu et al. provides a mechanistic basis for interoceptive interventions by identifying a shared neural pathway for interoception and anxiety. Using a novel paradigm that engages interoceptive awareness without requiring focused attention on a single organ, they found that both processes recruit overlapping circuits for bodily awareness. This neural overlap suggests that modulating activity in these shared pathways—for example, through techniques that alter interoceptive processing—could directly alleviate anxiety (2018).

Mindfulness‐Based Stress Reduction (MBSR) is one such intervention that targets this interoceptive‐anxiety nexus (Khalsa et al. 2018, Farb et al. 2015, Kabat‐Zinn 2005). It operates through bidirectional (bottom‐up and top‐down) modulation of bodily signals. A core component, the mindfulness body scan, trains individuals to sustain non‐judgmental attention on interoceptive sensations, beginning with the breath—a key psychosomatic indicator whose frequency is directly altered by anxiety (Weng et al. 2021, Homma and Masaoka 2008, Nardi et al. 2009). By practicing the voluntary regulation of attention and acceptance of sensations (including potential “threats”), patients can counteract maladaptive threat biases during early cognitive processing (Gan et al. 2022). Brief, accessible versions of this practice show particular promise for directly modulating the dysfunctional interoceptive inference cycles characteristic of anxiety disorders (Kabat‐Zinn 2005, Carmody and Baer 2009, Sauer‐Zavala et al. 2012, Beng et al. 2016).

In summary, building on the synthesis of evidence presented above, we propose that future research must establish a unified framework for developing precise interoceptive interventions. To this end, we advocate for a two‐pronged approach: 1) leveraging computational models to formalize the mechanisms of existing techniques (e.g., elucidating how body scans alter the precision weighting of bodily signals), and 2) employing rigorous experimental paradigms to validate their efficacy. A critical next step is to conduct double‐blind studies that test whether practices like the body scan directly remediate maladaptive interoceptive inference by providing counter‐evidence to threat priors. These studies should manipulate interoceptive‐exteroceptive signal relationships (e.g., using delayed cardiac visual feedback) to provoke and measure prediction errors in anxious individuals. Simultaneously, neurophysiological markers (e.g., heart‐evoked potentials, insula activity) must be integrated to identify objective neural signatures of interoceptive bias and its correction (Feinstein et al. 2018b, Hassanpour et al. 2017, Schulz et al. 2019). Ultimately, by grounding intervention research in this computationally informed framework, we can move beyond generic treatments and toward targeted therapies that recalibrate specific dysfunctional nodes within the interoceptive inference hierarchy, thereby significantly improving clinical outcomes for anxiety disorders.

## 6 Conclusions

Interoception, a key link between the body and mind, plays a crucial role in diagnosing and treating mental health conditions, especially anxiety disorders. The computational theory of interoception provides valuable insights into cognitive impairments in interoceptive functioning and the integrative regulatory role of interoception in these conditions. By applying computational models alongside techniques like body scans, researchers can assess interoceptive dysfunctions in anxiety patients. This integrative approach not only deepens our understanding of anxiety disorders but also facilitates the translation of behavioral and cognitive data into targeted interventions. Ultimately, such strategies may help patients more effectively manage both the physical and psychological challenges of anxiety.

## Author Contributions

**Zihan Lin**: conceptualization, methodology, visualization, writing – original draft, writing – review and editing. **Shiqi Liao**: conceptualization, methodology. **Shasha Zhu**: conceptualization, methodology. **Yuqing Zhao**: conceptualization, methodology. **Wen‐jing Yan**: writing – review and editing. **Ke Jiang**: writing – review and editing, supervision, project administration. **Kaiyu Qiu**: writing – review and editing.

## Funding

The authors have nothing to report.

## Conflicts of Interest

The authors declare no conflicts of interest.

## Data Availability

The authors have nothing to report.
